# Endotracheal Intubation Using the Macintosh Laryngoscope or KingVision Video Laryngoscope during Uninterrupted Chest Compression

**DOI:** 10.1155/2014/250820

**Published:** 2014-06-04

**Authors:** Ewelina Gaszynska, Tomasz Gaszynski

**Affiliations:** ^1^Department of Hygiene and Health Promotion, Medical University of Lodz, Poland; ^2^Department of Emergency Medicine and Disaster Medicine, Medical University of Lodz, Ulica Kosciuszki 4, 90-419 Lodz, Poland

## Abstract

*Objective.* Advanced airway management, endotracheal intubation (ETI), during CPR is more difficult than, for example, during anesthesia. However, new devices such as video laryngoscopes should help in such circumstances. The aim of this study was to assess the performance of the KingVision video laryngoscopes in a manikin cardiopulmonary resuscitation (CPR) scenario. *Methods.* Thirty students enrolled in the third year of paramedic school took part in the study. The simulated CPR scenario was ETI using the standard laryngoscope with a Macintosh blade (MCL) and ETI using the KingVision video laryngoscope performed during uninterrupted chest compressions. The primary endpoints were the time needed for ETI and the success ratio. *Results.* The mean time required for intubation was similar for both laryngoscopes: 16.6 (SD 5.11, median 15.64, range 7.9–27.9) seconds versus 17.91 (SD 5.6, median 16.28, range 10.6–28.6) seconds for the MCL and KingVision, respectively (*P* = 0.1888). On the first attempt at ETI, the success rate during CPR was comparable between the evaluated laryngoscopes: *P* = 0.9032. *Conclusion.* The KingVision video laryngoscope proves to be less superior when used for endotracheal intubation during CPR compared to the standard laryngoscope with a Mackintosh blade. This proves true in terms of shortening the time needed for ETI and increasing the success ratio.

## 1. Introduction

**Figure 1:**
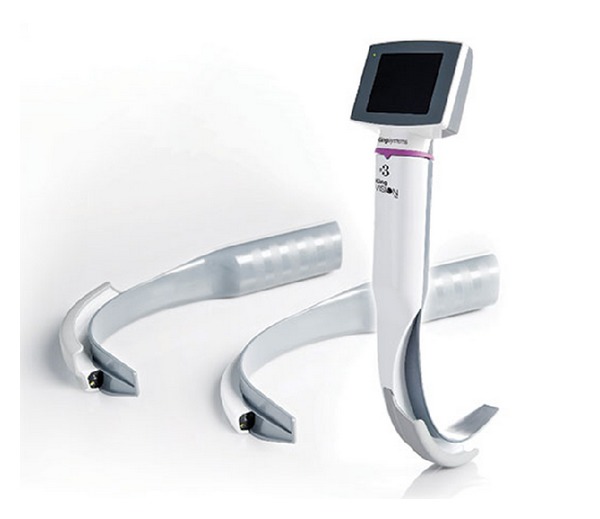
KingVision video laryngoscope (source: manufacturer marketing materials).

It is strongly advised by the European Resuscitation Council not to interrupt the chest compressions for prolonged instrumental airway management in order to maximize coronary and cerebral perfusion pressure [1]. Though, a very short pause in chest compressions is allowed for fast endotracheal intubation. Skilled operators should (remove even) try to secure the airway without interruption in chest compressions [1]. In previous reports, it was shown that chest compressions prolonged the time needed for successful endotracheal intubation [2]. There are several new devices for endotracheal intubation that were tested for effectiveness during cardiopulmonary resuscitation. Presently, the most common method of definite airway management is endotracheal intubation using the standard Macintosh blade (MCL). Chest compressions may decrease the laryngeal view during CPR; therefore, endotracheal intubation may be more difficult in these cases. The KingVision (King Systems, PN, USA) is a new video laryngoscope in which the view of glottis is displayed on a small monitor incorporated into the handle of the laryngoscope (Figure 1). It has single-use blades with or without a guide channel for the endotracheal tube. The advantage of the KingVision device is that the operator can look at the monitor making it possible to visualize the larynx from a certain distance without the necessity to closely approach the patient, similar to that of other video laryngoscopes. This proves helpful during resuscitative measures.

Another advantage of the KingVision is that its shape should allow for easier and less traumatic intubation. There are no studies on the use of the KingVision for resuscitation, so this is the first report evaluating the KingVision video laryngoscope in such settings.

We hypothesized that the KingVision video laryngoscope may provide shorter intubation times and/or higher intubation success rates during uninterrupted chest compressions.

The aim of this study was to assess the performance of the KingVision video laryngoscope in a manikin cardiopulmonary resuscitation (CPR) scenario. The primary endpoints were the time needed for ETI and the success ratio.

## 2. Methods

The study protocol was approved by the Medical University of Lodz Ethics Committee (Protocol number: RNN/607/10/KB, chairperson: Professor P. Polakowski, on 12 October, 2010). Thirty students enrolled in the third year of paramedic school took part in the study. The students had experience in endotracheal intubation (ETI) as they learned it during the course of their studies, which included performing intubation in a simulated scenario of cardiopulmonary resuscitation. All participants were informed about the purpose of the study and they signed a written consent for inclusion. They had standard training, of no less than 30 minutes, using the KingVision video laryngoscope on manikin models before the study. They also had the opportunity to intubate the same manikin that was used for the study as many times as they felt necessary to gain adequate skills in using the KingVision video laryngoscope. The simulated CPR scenario was ETI using the standard laryngoscope with a Mackintosh blade (MCL) and ETI using the KingVision. Airway management was performed during uninterrupted chest compressions on the immobile manikin Ambu MegaCode Man (Ambu, Holland) with normal airway patency (no difficult airway simulation) while lying on the ground. The devices used by participants to intubate the manikin were allocated based on envelope randomization. The investigator running the scenario told the participant when to make an attempt and recorded (using an IVT stopwatch, Conrad Electronic, Germany) the time elapsed from the moment the participant took hold of each device to effective manikin ventilation with a bag valve mask (BVM), confirmed by a volumeter on the manikin. A size three MCL blade or the KingVision video laryngoscope with an* e*-tube guide channel was used at random in each scenario. Every participant intubated the manikin only once. For each insertion, all airway devices and the manikin's airway were well lubricated in accordance with the instructions of the manufacturer. The internal diameter of the tracheal tube was 7.5 mm. The manikin was placed on the floor, and all trials were performed at the same level. One participant continued chest compressions while the other performed airway management. The frequency of chest compressions was 100/min, and the clock was used to keep it constant. The depth of chest compressions was confirmed by the electronic measurement of the Ambu MegaCode Man manikin system as 4-5 cm.

The primary endpoints were the time (mean and standard deviation) needed for successful intubation (TTI) in the first attempt for each laryngoscope and success rate of the first attempt related to time. During each attempt, the time elapsed was recorded by a single observer using the same stopwatch. A failed intubation attempt was defined as esophageal intubation or insertion attempt lasting longer than 60 seconds [3].

The statistical analysis was performed using the Statistica PL package. Statistical analysis was performed using the chi-square test for independent pairs with Yates correction if necessary. Mann-Whitney* U* test was used for nonpaired categorical and continuous data analysis, respectively. Repeated measure analysis of variance was used for TTI comparisons between the three attempts and each variable. Post hoc testing was performed using the Tukey HSD test. Kaplan-Meier curves were drawn and comparisons between groups were performed with the log rank test. *P* values lower than 0.05 were considered as statistically significant.

## 3. Results

**Figure 2:**
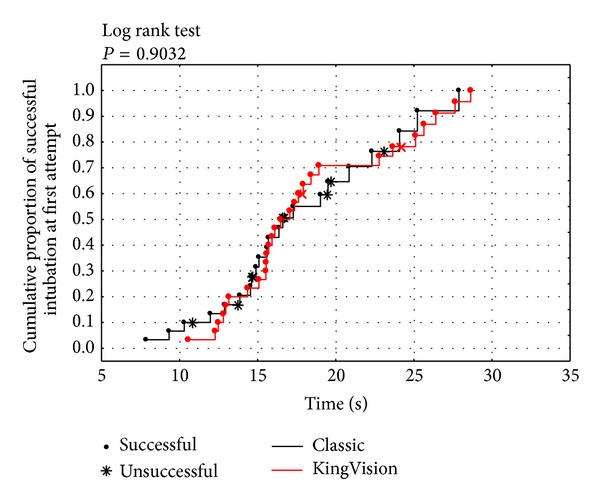
A comparison of cumulative success rate between KingVision video laryngoscope and Macintosh laryngoscopes.

Participants were students of the final (third) year of rescue medicine at the Medical University of Lodz. The mean age was 21 yrs, 18 men and 12 women. All participants were trained in advanced airway management including endotracheal intubation during their studies and gained qualifications required to perform endotracheal intubation in emergency situations. The mean time needed for intubation was similar for both laryngoscopes: 16.6 (SD 5.11, median 15.64, range 7.9–27.9) seconds versus 17.91 (SD 5.6, median 16.28, range 10.6–28.6) seconds for the MCL and KingVision, respectively (*P* = 0.1888). The success ratio on the first attempt of ETI during uninterrupted chest compressions was slightly better for the KingVision compared to the MCL: 93.3% versus 73.3% without significance (*P* = 0.0771; OR = 7, 95% CI 0.86–56.90). The cumulative success ratio of the first attempt of ETI during CPR was similar between evaluated laryngoscopes: *P* = 0.9032 (Figure 2).

## 4. Discussion

The European Resuscitation Council recommends tracheal intubation to secure a patient's airway during cardiopulmonary resuscitation as part of Advanced Life Support performed by well-trained professional rescuers. This gold standard is a procedure that requires highly qualified and experienced operators who may not be present during the first minute of the emergency. If tracheal intubation can be performed during uninterrupted chest compressions, this will sustain circulation during the procedure of intubation and may lead to successful resuscitation. In our study, the time needed for successful endotracheal intubation and cumulative success ratio were comparable between the KingVision video laryngoscope and the MCL.

This is the first report using the KingVision video laryngoscope during CPR but we may compare our results to the results of other researchers because they have evaluated devices similar in construction to the KingVision: Pentax-AWS and Airtraq. Such devices are from the group of devices equipped with a special channel for the endotracheal tube. Koyama et al. performed a study comparing three types of laryngoscopes on a manikin as to whether they enabled tracheal intubation while the manikin's chest was rhythmically compressed [4]. Thirty-five persons with little or no experience in intubation served as examinees. The laryngoscopes employed were a conventional Macintosh laryngoscope, a new video laryngoscope, Pentax-AWS, and an optic laryngoscope Airtraq. The success rates with the Pentax-AWS were significantly higher than those with the MCL or Airtraq and comparable to our results. In our previous study, we compared Airtraq and MCL in the same settings. We observed a longer time of ETI for Airtraq (21.8 s) and lower success rates (76.2%) [5]. This corresponds to the results of Koyama. Another study using the Pentax AWS during CPR was performed by Han et al. [6]. They compared the Pentax AWS and the MCL in three scenarios of CPR. Their results were as follows: the Pentax AWS had a 100% success rate with a time of ETI of 13.5 s, and MCL had 81.3% success rate with a time of ETI of 16.6 s. The successful clinical use of the Pentax AWS for ETI during CPR was described by Sadamori et al. [7]. Kim et al. evaluated whether chest compressions affected TTI using 3 laryngoscopes operated by 20 paramedic students: MCL, Glidescope, and Airway Scope [8]. They found that TTI was not significantly affected by chest compressions, but cumulative success rates related to TTI were significantly higher for video laryngoscopes. Our observations are similar for TTI. Study of Cho et al. confirms that use of video laryngoscopes (in this case Airway scope which is very similar in construction and use to KingVision) does not improve TTI [9]. In another study of Kim et al., researchers found out that chest compressions affect TTI performed by Pentax-AWS video laryngoscope and difference in TTI between Pentax-AWS and MCL is not significant in these conditions [10]. However, as a conclusion, Kim and Cho state that “Considering the lack of experience, video laryngoscopes may be useful adjuncts for intubation by experienced intubators during chest compressions.” Shin et al. came to different conclusions [11]. TTI in their study was significantly better for Pentax-AWS comparing to MCL during uninterrupted chest compressions.

Our study has several limitations. Firstly, it is performed on a manikin model, not on real patients. Due to technical and ethical issues, it may be difficult to perform a crossover study on human models. Although the study was on manikin models, it is still of value and suggests the ideal approach in real situations. Secondly, the number of tested participants is limited. The sample size could have been bigger but because of technical issues such as limited access to a larger number of working physicians at the time of conducting the study, this study as with many others is performed with similar numbers of participants. Finally, we admit that the chest compression scenario was always after performing the intubation on a manikin without CPR. This may influence the results; but in real training, the new devices are used on patients in non-life-threatening situations and after becoming familiar with new equipment which is used by physicians in emergency cases.

We did not calculate the sample size; however, based on similar published studies [8, 9], it was estimated that a group of 30 participants is sufficient to detect a difference in the TTI between evaluated devices.

While comparing the results, we may state that the KingVision video laryngoscope has a similar performance to the Pentax AWS during CPR.

## 5. Conclusion

In conclusion, within these limitations, based on the results of our observation, we conclude that the KingVision laryngoscope is a device less superior for endotracheal intubation during CPR compared to the standard laryngoscope with a Mackintosh blade in terms of shortening the time needed for ETI and increasing the success rate.

## References

[1] Hazinski MF, Nadkarni VM, Hickey RW, O’Connor R, Becker LB, Zaritsky A (2005). Major changes in the 2005 AHA guidelines for CPR and ECC: reaching the tipping point for change. *Circulation*.

[2] Gatward JJ, Thomas MJC, Nolan JP, Cook TM (2008). Effect of chest compressions on the time taken to insert airway devices in a manikin. *British Journal of Anaesthesia*.

[3] Gaszynski T, Gluszcz R, Dobielski P, Jakubiak J (2009). Guidelines for the procedure to be used in the instance of unexpected difficulties with intratrachealintubation: wytyczne postepowania w przypadku nieprzewidzianych trudnosci z wykonaniem intubacji dotcchawiczej. *Anest Inten Terap*.

[4] Koyama J, Iwashita T, Okamoto K (2010). Comparison of three types of laryngoscope for tracheal intubation during rhythmic chest compressions: a manikin study. *Resuscitation*.

[5] Gaszynski TM (2011). Comparison of tracheal intubation by the Mackintosh laryngoscope and AirTraq during chest compression: a mannequin study. *European Journal of Anaesthesiology*.

[6] Han SK, Shin DH, Choi PC (2010). Utility of the Pentax-AWS without interruption of chest compression: Comparison of the Macintosh laryngoscope with the Pentax-AWS in manikin model. *Resuscitation*.

[7] Sadamoria T, Kusunokib S, Ishida M, Otania T, Tanigawaa K (2008). Video laryngoscopy for emergency tracheal intubation during chest compression. *Resuscitation*.

[8] Kim YM, Kang HG, Kim JH, Chung HS, Yim HW, Jeong SH (2011). Direct versus video laryngoscopic intubation by novice prehospital intubators with and without chest compressions: A pilot manikin study. *Prehospital Emergency Care*.

[9] Cho J, Chung HS, Chung SP, Kim YM, Cho YS (2010). Airway scope vs Macintosh laryngoscope during chest compressions on a fresh cadaver model. *American Journal of Emergency Medicine*.

[10] Kim YM, Kim JH, Kang HG, Chung HS, Yim HW, Jeong SH (2011). Tracheal intubation using Macintosh and 2 video laryngoscopes with and without chest compressions. *American Journal of Emergency Medicine*.

[11] Shin DH, Choi PC, Han SK (2011). Tracheal intubation during chest compressions using Pentax-AWS, GlideScope, and Macintosh laryngoscope: a randomized crossover trial using a mannequin. *Canadian Journal of Anesthesia*.

